# Circulating Tumor Cell Migration Requires Fibronectin Acting through Integrin B1 or SLUG

**DOI:** 10.3390/cells9071594

**Published:** 2020-07-01

**Authors:** Jeannette Huaman, Olorunseun O. Ogunwobi

**Affiliations:** 1 Department of Biological Sciences, Hunter College of The City University of New York, New York, NY 10065, USA; JHUAMAN@genectr.hunter.cuny.edu; 2 Department of Biology, The Graduate Center of The City University of New York, New York, NY 10016, USA; 3 Department of Medicine, Weill Cornell Medicine, New York, NY 10065, USA

**Keywords:** fibronectin (FN1), cancer cell migration, circulating tumor cells (CTCs), integrin B1 (ITGB1), SLUG (SNAI2), metastasis

## Abstract

Fibronectin (FN1) is an extracellular matrix protein gaining increasing attention for its multifaceted roles in cancer progression. Using our recently established circulating tumor cell (CTC) lines, we had demonstrated increased FN1 expression and enhanced migration in CTC lines, in comparison to primary tumor cell lines. Whether increased FN1 expression is directly required for CTC migration, and the specific role of FN1’s regulation of integrin B1 (ITGB1) and SLUG (SNAI2) in CTC migration remains unclear. Here, for the first time, we report that the knockdown of FN1, ITGB1, or SLUG expression in CTCs leads to a significant decrease in CTC migration. Knocking down two or all three of these proteins simultaneously did not further inhibit migration. We observed a corresponding increase in CTC migration when recombinant FN1 was added to CTCs. This effect was significantly impeded by prior knockdown of ITGB1 or SLUG. Using knock down experiments and western blotting analysis, we confirmed FN1’s regulation of ITGB1 and SLUG to occur via two separate, independent pathways. Consequently, we can conclude that FN1-dependent enhanced migration of CTCs requires downstream signaling through either ITGB1 or SLUG and that FN1 regulation of ITGB1 and SLUG may have important implications for cancer progression and metastasis.

## 1. Introduction

The extracellular matrix (ECM) is a network rich in proteins and carbohydrates that supports tissue structure, promotes cell communication, and enables cell adhesion [1,2]. During cancer progression, the ECM is subject to considerable alterations. Fibronectin (FN1) is a critical component and regulator of the ECM [3,4,5,6]. Interestingly, FN1 is overexpressed in a variety of cancers. Moreover, increased FN1 expression in tumor tissue has been linked to a poorer prognosis in cancer patients [7,8,9,10,11,12,13,14]. Since its discovery in 1974, FN1 has gained recognition for being a critical player in multiple aspects of cancer progression, including tumor cell migration, invasion, and metastasis [3,5,15,16,17,18]. 

Metastasis accounts for over 90% of cancer mortalities worldwide [19,20,21,22]. A critical step in the metastatic cascade is the detachment of cancer cells from the primary tumor and their subsequent migration and intravasation into the bloodstream. These cells, known as circulating tumor cells (CTCs), can help us better understand critical aspects of the metastatic cascade [23,24,25]. It is, however, challenging to study CTCs as they are found in small numbers in the blood [26,27,28]. To address this challenge, we have expanded CTCs into cell lines. As previously reported [29], we successfully established three CTC lines: two from a syngeneic mouse model of hepatocellular carcinoma (HCC) and one from a human xenograft mouse model of castration-resistant prostate cancer (CRPC). Both HCC and CRPC are examples of cancers that frequently advance, metastasize, are difficult to treat, and result in death [30,31,32,33,34,35,36,37,38]. 

Functional characterization of our established cell lines revealed that CTCs display greater migration in comparison to primary tumor cells. Moreover, CTCs demonstrated increased fibronectin (FN1), integrin B1 (ITGB1), and SLUG expression. In the present study, we sought to determine whether the enhanced migration observed in CTCs was due to increased expression of FN1, ITGB1, or SLUG. Further, we investigated whether FN1 regulation of ITGB1 and SLUG is required for CTC migration. Finally, we examined whether FN1 regulation of ITGB1 is dependent on SLUG, or whether FN1 regulation of SLUG is dependent on ITGB1, thereby potentially linking these three molecules in the same pathway. Altogether, our study reveals novel molecular mechanisms by which FN1 may regulate CTC migration, and further highlights the importance of FN1 in cancer progression and metastasis.

## 2. Materials and Methods

### 2.1. Cell Lines and Cell Culture

A detailed description of how CTC lines were established from both a syngeneic mouse model of HCC and a human xenograft mouse model of CRPC can be found in [29]. This resulted in the following CTCs which were used in this study: CBOH4 and CBOH9 (for our HCC models) and C22OH (for our CRPC model).

CBOH4 and CBOH9 cell lines were cultured in DMEM media. Media was supplemented with 1% penicillin/streptomycin, 10% fetal bovine serum, and L-glutamine. Upon reaching 75–80% confluency, cells were passaged using 0.25% trypsin.

C22OH cell line was cultured in RPMI media. Media was supplemented with 1% penicillin/streptomycin and 10% fetal bovine serum. Upon reaching 75–80% confluency, cells were passaged using 0.05% trypsin. All CTCs were maintained in a 5% CO_2_, 37 °C incubator. 

### 2.2. Transfection of Cells with siRNAs

Cells were seeded in 6-well plates. Upon reaching 60–70% confluency, cells were transfected with 10nM of appropriate siRNAs: siFibronectin (Santa Cruz Biotechnology, Dallas, TX, USA, cat#: sc-29315), siIntegrinB1 (Santa Cruz Biotechnology, Dallas, TX, USA, cat#: sc-35674), siSLUG (Santa Cruz Biotechnology, Dallas, TX, USA, cat#: sc-38393), or a non-targeting scramble control (Sigma, St. Louis, MO, USA) using Lipofectamine RNAiMAX (Thermo Fisher Scientific Inc., Wilmington, DE, USA) diluted in Opti-MEM (ThermoFisher Scientific Inc., Wilmington, DE, USA). Final concentration of siRNAs per well was 25 pmol. Cells were incubated for 24 h after which they were harvested.

### 2.3. Treatment of Cells with Recombinant Fibronectin Protein

Cells were seeded in 6-well plates. Upon reaching 60–70% confluency, cells were treated with Recombinant Fibronectin (R&D Systems, Minneapolis, MN, USA). Final concentration of recombinant protein per well was 2 μg/mL.

### 2.4. Migration Assays

Migration was evaluated in CBOH4 and CBOH9 via wound healing assays. Cells were seeded in 6-well plates and permitted to reach 90–100% confluency. A plastic tip (1 mm thick) was used to create a wound in the monolayer of cells within each well. Cells were washed with 1× PBS and incubated with media. Wounds were measured at Time 0. Experiments were terminated when wounds of one cell line or condition closed. Wound areas were measured and quantified using Motic AE software. At least 30 fields per cell line/condition were recorded. Three independent experiments were performed. Images of cells were taken using the Motic AE30 Nikon Inverted Microscope. 

C22OH migration was evaluated using transwell migration assays. This assay was used instead of the wound healing migration assay because it took beyond 3 days to close wounds in this CTC line. Wound healing assays spanning this amount of extended time have the possibility of introducing other factors besides migration to close wounds. To avoid this, the transwell migration assay was used instead. Cells were placed on top of an 8 μm pore transwell chamber (Greiner Bio-one, Kremsmünster, Austria, cat #: 662 638) in serum-free media. Below transwell chambers, regular media was placed to act as an attractant. After 24 h, cells that had migrated through the pores and adhered to the bottom of the transwell chambers were rinsed with 1× PBS, fixed with paraformaldehyde and treated with methanol. Cells were subsequently stained with trypan blue, placed on a slide, and viewed using the Motic AE30 Inverted Microscope. All migration assays were performed 3 times. The following equations were used to determine percentage increases or decreases in migration. Percentage (%) Increase = (Amt of increase/Original Amt) × 100; Percent (%) Decrease = (Amt of Decrease/Original Amt) × 100.

### 2.5. Protein Extraction and Western Blotting

Cells were treated with RIPA lysis buffer (Amresco, Cleveland, OH, USA, cat#: N653), supplemented with 10× protease inhibitors (Thermo Fisher Scientific, Rockford, IL, USA, cat#: 88665) and 100 mM PMSF (Amresco, Cleveland, OH, USA, cat#: M145). Bradford Assay was used to determine protein concentration, using Bio-Rad’s Protein Assay Dye Reagent Concentrate. For Western blots, 30 μg of protein were run on precast SDS-PAGE gels and transferred onto nitrocellulose membranes. Blocking of membranes was performed using 5% BSA in TBS-T for 1 h at room temperature. Membrane were incubated with primary antibodies overnight at 4 °C. After incubation, membranes were washed with 1× TBS-T, incubated with secondary antibodies for 1 h, washed, and processed using the LI-COR Odyssey CLx imager with infrared fluorescence. The following primary antibodies used: integrin B1: 4706S (1:500; Cell Signaling, MA, USA), SLUG: 9585S (1:500; Cell Signaling, Danvers, MA, USA), and alpha-tubulin: sc-32293 (1:500; Santa Cruz Biotechnology, Dallas, TX, USA). Secondary antibodies used were anti-rabbit: 925-32211 (1:15,000; LI-COR, Lincoln, NE, USA), and anti-mouse: 925-32210 (1:15,000; LI-COR, Lincoln, NE, USA). Image J software was used to analyze and quantify western blot bands.

### 2.6. Statistical Analyses

Data from three independent experiments were collected and used to construct corresponding graphs which were presented as mean + standard error (SEM) of the mean. Statistical significance for all experiments were evaluated using Student’s *t* test. The *p* values < 0.05 were deemed significant.

## 3. Results

### 3.1. FN1, ITGB1, and SLUG Play Equally Important Roles in Regulating Circulating Tumor Cell (CTC) Migration 

**Figure 1:**
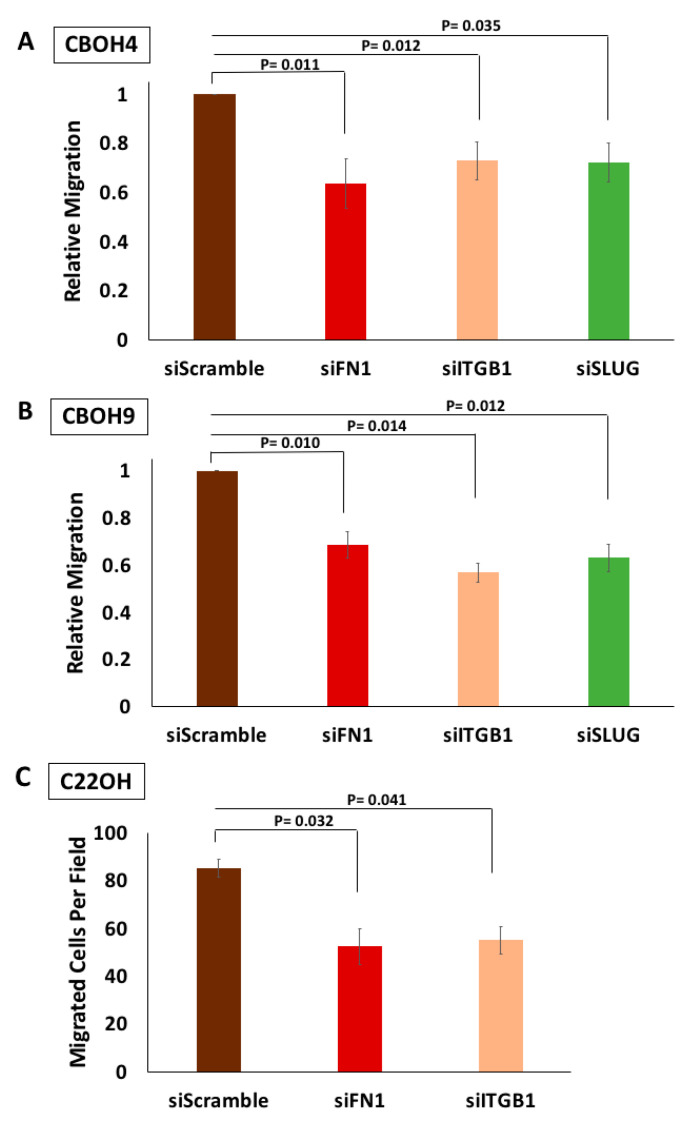
Circulating tumor cell (CTCs) demonstrate decreases in migration upon FN1, ITGB1, and SLUG transient knockdown. (**A**) Knockdown of FN1, ITGB1, and SLUG and their effects on CBOH4 migration. (**B**) Knockdown of FN1, ITGB1, and SLUG and their effects on CBOH9 migration. (**C**) Knockdown of FN1 and ITGB1 and their effect on C22OH migration. Transient transfections, using a final concentration of 25 pmol, were performed for a total of 12 h until wounds closed for at least one condition in CBOH4 and CBOH9. Transwell migration assays for C22OH were carried out for 24 h. All experiments were performed three times. Statistical analysis was performed using *t*-test, where *p* < 0.05 was deemed significant.

We previously identified CTCs to have increased FN1 expression as compared to primary tumor cells [29]. In agreement with FN1’s established role in migration [5,16], CTCs also exhibited enhanced migration. Interestingly, we were the first to report FN1’s regulation of ITGB1 and SLUG in CTCs [29]. Both ITGB1 and SLUG are molecules with previous implications in migration as well [39,40,41,42,43,44,45,46]. To confirm whether FN1 and its downstream effectors, ITGB1 and SLUG, are necessary for CTC migration, we performed transient knockdowns of FN1, ITGB1, and SLUG. As shown in Figure 1A,B, knockdown of all three molecules caused a significant decrease in migration in comparison to siScramble. CBOH4 exhibited a 36% decrease in migration when FN1 was knocked down, followed by a 27% and 28% decrease when ITGB1 and SLUG were knocked down. Similarly, CBOH9 migration lowered by 31% when FN1 was knocked down, followed by 43% and 37% decreases upon ITGB1 and SLUG knockdown. Unfortunately for C22OH, our single CRPC CTC line, we were unable to get a successful SLUG knockdown. Nevertheless, as shown in Figure 1C, migration was reduced by 39% and 35% when FN1 and ITGB1 were knocked down, respectively.

To determine whether knocking down multiple players simultaneously elicits a greater reduction in migration, we performed transient double and triple knockdowns. For all three CTC lines, we observe no additional advantage to knocking down both FN1 and ITGB1, FN1 and SLUG, or ITGB1 and SLUG (see Supplementary Figure S1A). Likewise, knocking down all three molecules (FN1, ITGB1, and SLUG) revealed no further decrease in migration when compared to single protein knockdowns. For all migration assays, student’s *t*-test analyses were performed to compare the CTC migration in cells receiving single/double/triple knockdowns with cells receiving siScramble. Resulting *p* values reveal all migration decreases to be significant, whether single, double, or triple knockdowns. 

### 3.2. ITGB1 Does Not Regulate SLUG Expression Levels in CTCs

**Figure 2:**
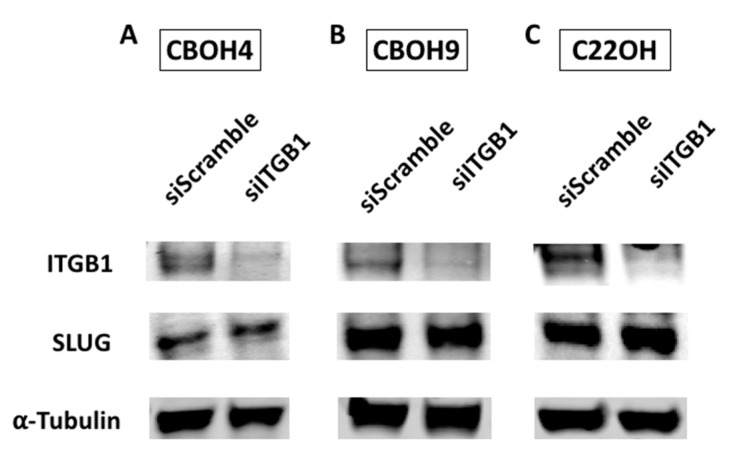
Knockdown of ITGB1 does not affect SLUG expression in CTCs. Western blot analysis of SLUG protein expression levels when ITGB1 is transiently knocked down in (**A**) CBOH4, (**B**) CBOH9, and (**C**) C22OH. Expression was normalized against alpha-tubulin. Experiments were carried out at least 3 times.

Previously, we published about FN1’s regulation of both ITGB1 and SLUG [29]. To determine whether ITGB1 and SLUG work in the same pathway, and whether ITGB1 regulates SLUG, we transiently knocked down ITGB1 and looked at resulting effects on SLUG expression. As seen in Figure 2A–C, SLUG revealed no change when ITGB1 was knocked down in all three CTC lines. From this observation, we can conclude that ITGB1 does not appear to regulate expression levels of SLUG or work in the same pathway in this manner.

### 3.3. FN1’s Effects on CTC Migration Requires ITGB1 Expression

**Figure 3:**
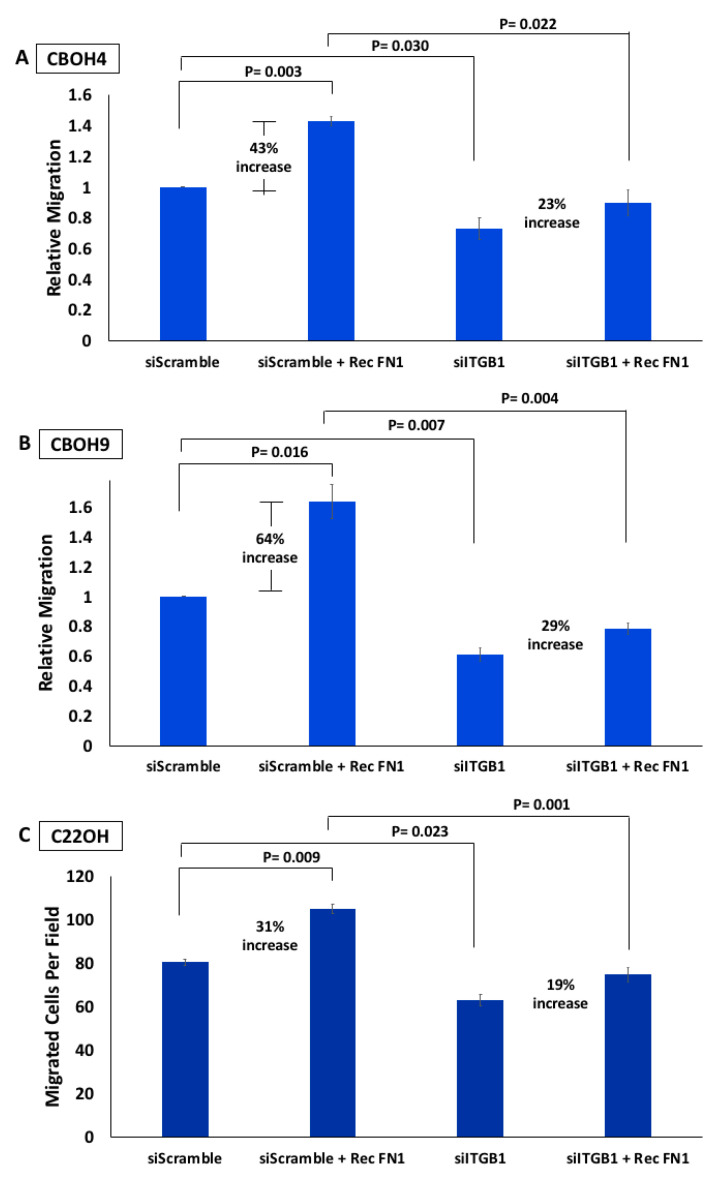
Recombinant FN1 is enough to induce greater CTC migration but not sufficient to completely rescue migration deficits when ITGB1 is knocked down. Recombinant FN1 was added to CTCs that were treated concurrently with either siScramble or siFibronectin. Migration was then assessed via wound healing assays performed on (**A**) CBOH4 and (**B**) CBOH9 CTCs, or transwell migration assays performed on (**C**) C22OH CTCs. Data provided on graphs are presented as mean ± standard error of the mean (SEM); *n* = 3. Statistical analysis was performed using *t*-test, where *p* < 0.05 was deemed significant.

To further elucidate the molecular mechanisms of our proposed FN1/ITGB1 cell migration pathway, we investigated the effects of adding FN1 to CTCs. We wanted to determine whether FN1 required ITGB1 to enhance CTC migration. To do this, CTCs were either transfected with siScramble or siITGB1 and then treated with recombinant FN1. Migration assays were performed to see whether recombinant FN1 would be enough to rescue dampened migration in CTCs whose ITGB1 had been knocked down. As shown in Figure 3A, recombinant FN1 significantly increased migration by 43% when added to CBOH4 that had been transfected with siScramble. This is in agreement with the literature on FN1’s promotion of migration. However, when recombinant FN1 was added to CBOH4 cells that previously demonstrated a 27% decrease in migration when transfected with siITGB1, the addition of FN1 was not enough to completely rescue the observed deficits in migration nor elicit as strong a migration increase when ITGB1 is otherwise present. 

Similarly in Figure 3B, we observed recombinant FN1’s ability to increase migration by 64% in CBOH9 transfected with siScramble. However, the addition of recombinant FN1 was not enough to completely rescue migration for CBOH9 cells previously transfected with siITGB1 and demonstrating a 39% decrease in migration. Instead, FN1 is only able to increase migration by 29%.

Finally, in our C22OH cell line, the addition of recombinant FN1 also increased migration by 31% in those cells transfected with siScramble but not siITGB1. C22OH cells transfected with siITGB1 exhibited a significant decrease in migration by 22% that was unable to be salvaged completely by the addition of recombinant FN1 (see Figure 3C).

From these results, we can conclude that FN1’s promotion of enhanced migration relies on the presence and function of ITGB1. While we did not test recombinant FN1’s ability to rescue migration in siFN1-treated cells, we observed a significant increase in migration when recombinant FN1 is added to cells. This supports FN1’s ability to significantly enhance migration if they had been treated with siFN1, as well as the requirement for ITGB1 to maximally enhance CTC migration.

### 3.4. SLUG Does Not Regulate ITGB1 Expression Levels in CTCs

**Figure 4:**
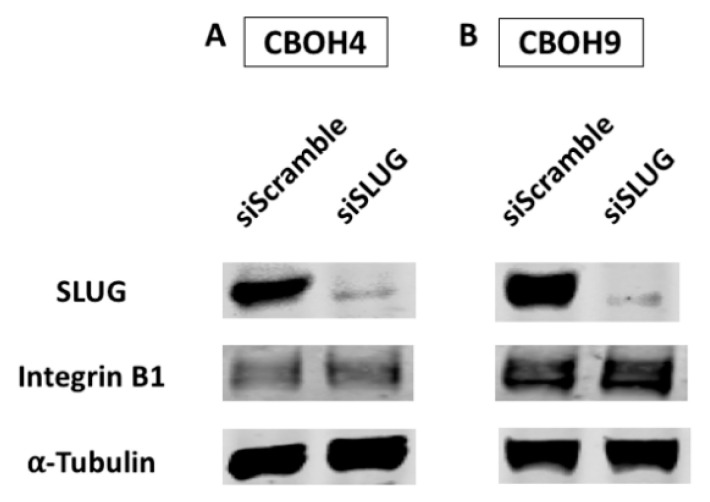
Knockdown of SLUG does not affect ITGB1 expression in CTCs. (**A**,**B**) Western blot analysis of ITGB1 protein expression levels when SLUG is transiently knocked down in CBOH4 and CBOH9. Expression was normalized against alpha-tubulin. Experiments were performed at least three times.

Having shown ITGB1 does not regulate SLUG expression, we assessed whether SLUG could regulate ITGB1 expression in CTCs. To determine this, we transiently knocked down SLUG using siRNAs. As shown in Figure 4A,B, ITGB1 protein expression was not affected by knocking down SLUG in either CBOH4 or CBOH9. Again, successful knockdown of SLUG was not effectively accomplished in our C22OH cell line, therefore this data is unavailable. However, we can still conclude that in the two CTC lines in which a successful SLUG knockdown was observed, SLUG does not appear to regulate ITGB1 in the same FN1-induced pathway. 

### 3.5. FN1’s Effects on CTC Migration Requires SLUG Expression

**Figure 5:**
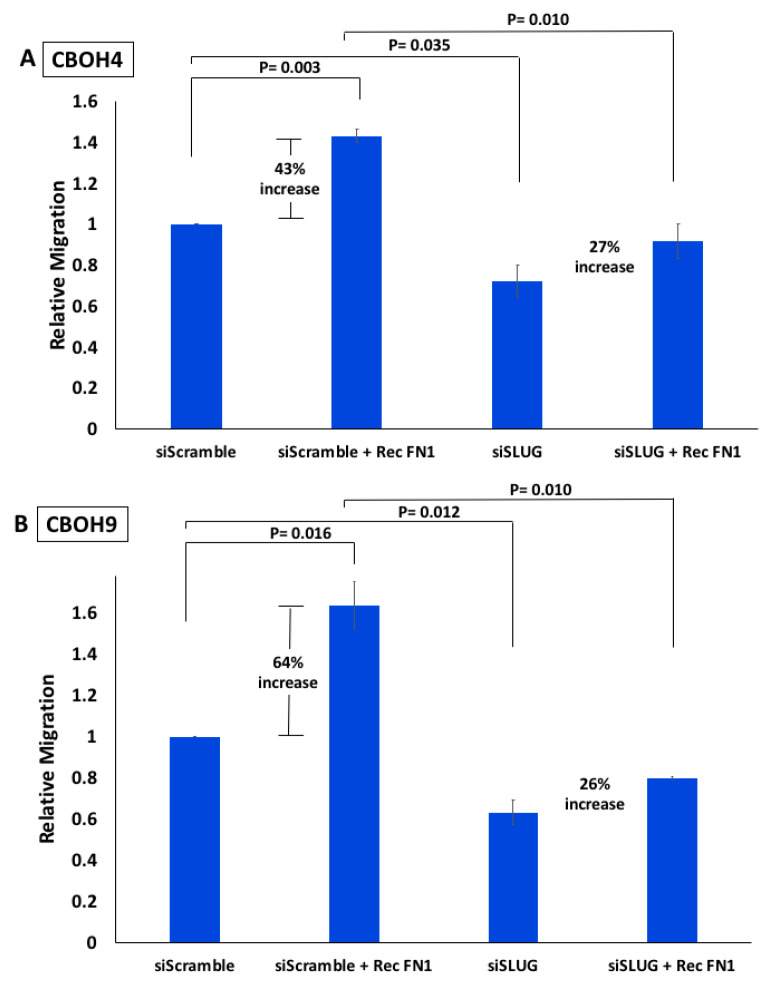
Recombinant FN1 is enough to induce greater migration in CTCs but not sufficient to completely rescue deficits in migration when SLUG is knocked down. Recombinant FN1 was added to CTCs that were treated concurrently with either siScramble or siSLUG. Migration was then assessed via wound healing assays performed on (**A**) CBOH4 and (**B**) CBOH9. Data are presented as mean ± standard error of the mean (SEM); *n* = 3. Statistical analysis was performed using *t*-test, where *p* < 0.05 was deemed significant.

Based on our observations, we evaluated whether FN1 requires SLUG for enhancing migration in CTCs. In agreement with its established role in the literature, FN1 is able to significantly increase migration by 43% when added to CBOH4 transfected with siScramble (See Figure 5A). However, when added to CBOH4 that previously demonstrated a 28% decrease in migration upon ITGB1 knockdown, recombinant FN1 was unable to fully rescue impaired CTC migration. 

We observed a similar result in Figure 5B. When FN1 is added to CBOH9 previously transfected with siScramble, we observed a 64% increase in migration. However, in cells that were transfected with siSLUG and demonstrated a 37% decrease in migration, FN1 was not sufficient to completely rescue migration. From these results, we can conclude that FN1’s ability to promote enhanced CTC migration relies on the presence and function of SLUG.

## 4. Discussion

FN1 is a matrix glycoprotein with a number of roles in human health and disease, including embryonic development, tissue regeneration, cell growth, migration, and tumorigenesis [4,5,6,15,16]. FN1’s ability to carry out its diverse functions depends on the various molecules it interacts with, including integrin transmembrane cell receptors and the ECM. Interestingly, upregulation of FN1 has been observed in a variety of cancers [7,8,9,10,11,12,13,14]. Nevertheless, a better understanding of how FN1 promotes tumorigenesis is still needed.

While a number of studies highlight FN1’s aberrant expression in tumor tissue for diagnostic and therapeutic targeting purposes [15,17,18], our work suggests exciting new mechanisms by which FN1 can promote cancer. We were particularly interested in studying the role of overexpressed FN1 in CTCs. The objective of our study was to determine how FN1 may enhance CTC migration by regulating ITGB1 and SLUG. We previously observed that in comparison to primary tumor cells, CTCs were more migratory and expressed greater levels of FN1, as well as its downstream effectors ITGB1 and SLUG [29]. We now demonstrate the substantial contributions of each of these molecules to CTC migration. Single knockdowns of FN1, ITGB1, and SLUG significantly lowered migration by as much as 43%, while double and triple knockdowns conferred no additional decrease in CTC migration. The efficiency and specificity of our knockdowns were verified and can be seen in Supplementary Figures S2 and S3.

We were also interested in establishing whether FN1’s regulation of ITGB1 and SLUG were 2 distinct pathways or whether they cooperated upstream or downstream of one another. Previous work done by others suggested they could work in the same pathway. Desgrosellier et al. [47], for example, demonstrated integrin B3’s ability to regulate SLUG in the neoplastic mammary gland. Furthermore, ITGB1 has been reported to activate TGFB1 [48,49], which itself has been observed upstream of SLUG [50]. Therefore, it is conceivable that FN1’s regulation of ITGB1 may control SLUG and affect CTC migration in this manner. To this end, we knocked down ITGB1 in our CTC lines and assessed SLUG expression. No change was observed. Alternatively, we tested whether SLUG was upstream of ITGB1. This too was plausible since others have reported SLUG to regulate integrins in keratinocytes [51], wound healing in the gut [52], and in ovarian cancer cells [53]. However, SLUG knockdown yielded no change in ITGB1 in our CTC models of HCC and CRPC. Consequently, we believe that due to different tumor microenvironments and active signaling pathways occurring in these distinct systems, SLUG may not be part of the same pathway as ITGB1 in CTCs of hepatic or prostate origin. These findings are important because we believe they depict yet another interesting scenario in which FN1 may exert its effects to enhance CTC migration independently via ITGB1 or SLUG.

FN1’s regulation of ITGB1 and SLUG further evoked questions of whether these molecules were necessary for FN1 to increase migration. CTCs treated with recombinant FN1 with no previous knockdown (siScramble only) exhibited a robust increase in CTC migration. However, when ITGB1 or SLUG were knocked down, the addition of recombinant FN1 proved incapable of eliciting the same prominent migratory increase. Only a partial recovery in migration was observed in most cases. We observed that knock down of ITGB1 or SLUG impaired FN1’s ability to enhance migration by as much as 50%. We therefore concluded that these two molecules are needed for FN1 to optimally enhance CTC migration. 

Our work is noteworthy in that it is one of the few in the FN1 oncogenic field [29,54,55,56,57,58] to highlight FN1’s active regulation of molecules to impact cancer cell migration rather than focus on its connecting role in the ECM. A bulk of oncogenic studies have focused on FN1’s intriguing overexpression in tumor tissue and its correlation with a poorer prognosis [5,15,17]. We are now addressing how FN1 may be doing so via the elucidation of novel pathways or molecules it regulates. Furthermore, we are among the first to our knowledge to establish these finding in CTCs. Several studies have described increases in FN1 expression in CTCs as a marker of epithelial-to-mesenchymal transition (EMT) [59,60,61,62] or CTCs benefiting from stromal FN1 produced by neighboring cells [63]. However, we now show that FN1 expression by CTCs plays a fundamental role in enhancing their own ability to migrate faster. 

**Figure 6:**
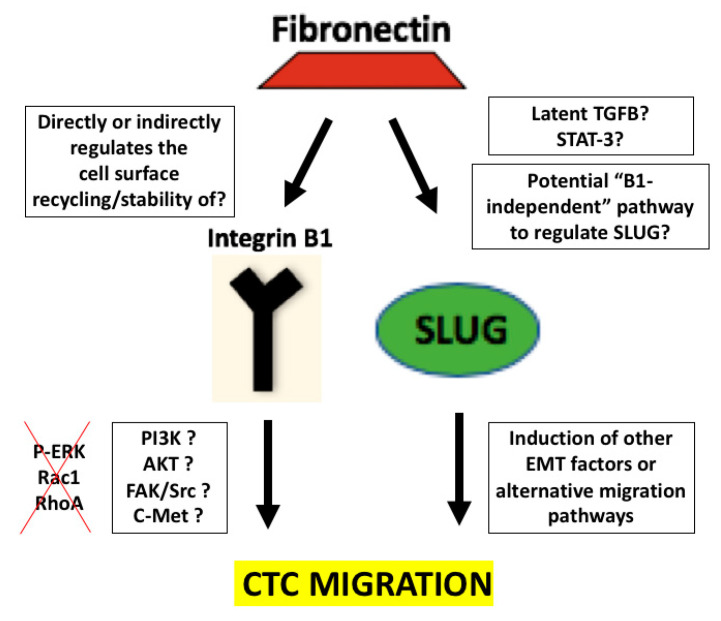
Proposed model of how FN1 overexpression in CTCs contributes to their enhanced migration capability. It has previously been observed that greater levels of FN1 expression in tumors has been correlated with poorer patient survival. Interestingly, our established CTC lines demonstrated a pronounced increase in FN1 as compared to our primary tumor cell lines. We have also previously reported that FN1 regulates both ITGB1 and SLUG. In this paper, we now show that FN1 regulates ITGB1 and SLUG via two separate and independent pathways. Furthermore, both ITGB1 and SLUG are necessary for FN1-mediated migration. This figure contains both confirmed data, as well as areas still in need of research.

In summary, we propose the following model, as shown in Figure 6. We propose that FN1 expression by CTCs is an important contributing mechanism for CTC migration. Furthermore, we propose that FN1’s regulation of ITGB1 and SLUG occur via two separate and independent pathways by which greater CTC migration is accomplished. Finally, we establish FN1’s requirement of ITGB1 and SLUG to maximally increase migration. Notwithstanding, our work leaves room for further investigation into several areas. For example, does FN1 regulate ITGB1 expression by affecting its stability or recycling it to the cell surface? Furthermore, what lies downstream of ITGB1 and SLUG? While we assessed the expression of several well-known molecules implicated in cancer cell migration including P-ERK, RhoA, and Rac1 [64,65,66,67,68,69], none of them were affected upon ITGB1 and SLUG knockdown in our CTC model (see Supplementary Figures S4 and S5). Notwithstanding, there are a number of other molecules that we have not tested but have been reported by others to be regulated by ITGB1 and SLUG, with roles in metastasis and invasive behavior [15,70,71,72,73]. Finally, FN1’s regulation of SLUG needs to be further delineated. Previous work by others show FN1’s ability to regulate latent TGFB1 which eventually gets activated during tumorigenesis [74,75]. Since TGFB1 has been known to be upstream of and work in coordination with SLUG to induce its effects on EMT [50,76], perhaps this is the way in which FN1 regulates SLUG to induce greater CTC migration.

Future relevant work may also include the determination of whether FN1 is acting via intracellular regulation of molecules on their way to the cell surface, or via the secretion and autocrine binding of FN1 to its own receptors or in a paracrine fashion affecting nearby tumor cells. Tagging FN1 and monitoring it via live cell imaging could help answer this question. Furthermore, these results should be followed up with experiments in 3D culture and in mice to gain a better understanding of CTCs in an in-vivo setting. However, it is important to note these experiments were specifically performed using CTCs obtained from mice as a way to expand this limited resource that exists in small quantities in the blood [26] but can reveal much about cells on their way to metastasize to other parts of the body. Additionally, the successful establishment of long-term CTC lines has been accomplished by only a few groups, and cells obtained in this manner have already been used to learn more about select metastasizing cell populations [77,78,79,80]. As reported previously, no obvious loss in plasticity has been observed in our CTC lines over the course of the number of passages used in our experiments [29]. Our CTC model represents a credible way to study mechanisms of migration in CTCs and how they contribute to cancer progression. While more work remains to be done to further understand the complexity of FN1 mechanisms, our study opens doors to the notion that FN1 works as more than just a glycoprotein connecting cells to the ECM, but as an active regulator of proteins to enhance CTC migration. 

## 5. Conclusions

In conclusion, we have investigated two novel pathways involving FN1 and further examined their molecular dynamics and effects on CTC migration. To the best of our knowledge, we were the first to report FN1’s regulation of ITGB1 and SLUG in CTCs. Now we report FN1’s regulation of both of these molecules working via two separate and independent pathways. Furthermore, all three molecules, FN1, ITGB1, and SLUG, were shown to contribute equally to CTC migration. Double and triple knockdowns did not confer any augmented decrease in migration when compared to single knockdowns. Finally, ITGB1 and SLUG were shown to be necessary for FN1 to exert its robust effects in promoting migration. While P-ERK, Rac1, and RhoA were not regulated by FN1, ITGB1, and SLUG in our CTC model, there remains ample opportunity to continue the investigation of what lies downstream of these two FN1 pathways and how exactly FN1 is regulating ITGB1 and SLUG—at the level of transcription or affecting its protein stability. Nevertheless, our work is important because it highlights new and unexpected ways in which greater FN1 produced by CTCs may enhance their tumorigenic properties. Moreover, elucidation of these two novel pathways allows for a better understanding of how FN1 works and how their dysregulation can potentially contribute to a poorer prognosis and metastatic spread in cancer patients. 

## Supplementary Materials

The following are available online at https://www.mdpi.com/2073-4409/9/7/1594/s1, Figure S1: CTC migration is inhibited by knockdown of FN1, ITGB1, and SLUG, Figure S2: Specific siRNA-mediated knockdowns of FN1, ITGB1, and SLUG, Figure S3: Confirmation of double and triple knockdown specificity and exclusion of off-target knockdown effects, Figure S4: Knockdown of ITGB1 does not affect P-ERK, ERK, Rac1, or RhoA protein expression, Figure S5: Knockdown of SLUG does not affect P-ERK, ERK, Rac1, or RhoA protein expression.
