# Supplementary material for: Circulating Tumor Cell Migration Requires Fibronectin Acting through Integrin B1 or SLUG
Source: Cells. 2020 Jul 1;9(7):1594. doi: 10.3390/cells9071594 (PMC7408126; doi:10.3390/cells9071594)
Supplement: Supplementary file 1 [file cells-09-01594-s001.pdf]

cells

MDPI

Supplementary Information

Circulating tumor cell migration requires fibronectin acting through integrin B1 or SLUG

Jeannette Huaman [1,2], **Olorunseun O. Ogunwobi** [1,2,3]*

[1] Department of Biological Sciences, Hunter College of The City University of New York, New York, NY 10065, USA
[2] Department of Biology, The Graduate Center of The City University of New York, New York, NY, 10016, USA
[3] Department of Medicine, Weill Cornell Medicine, New York, NY, 10065

*Correspondence: ogunwobi@genectr.hunter.cuny.edu

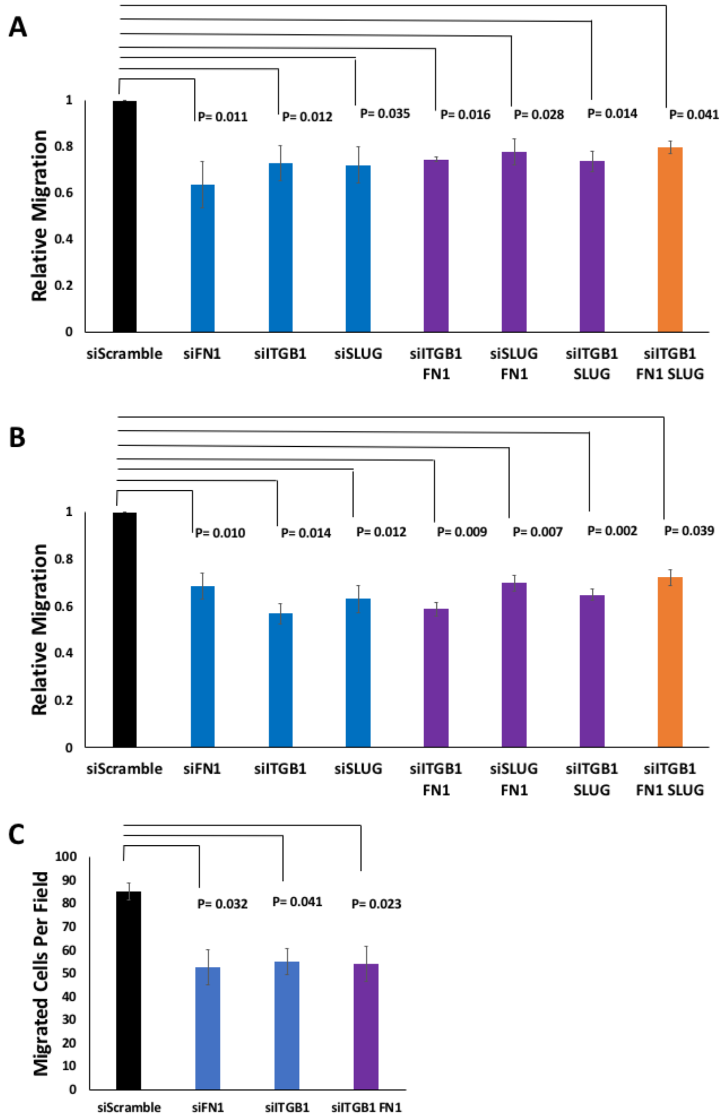

Figure S1: CTC migration is inhibited by knockdown of FN1, ITGB1, and SLUG. A) Combination of different knockdowns of FN1, ITGB1, and SLUG and their effects on CBOH4 migration. **B)** Combination of different knockdowns of FN1, ITGB1, and SLUG and their effects on CBOH9 migration. **C)** Combination of different knockdowns of FN1 and ITGB1 and their effect on C22OH migration. Transfections using a final concentration of 25pmol were performed.

Wound healing migration assays for CBOH4 and CBOH9 were performed for 12 hours until wound closure occurred for at least one condition. Transwell migration experiments for C22OH were carried out for 24 hrs. All experiments were performed 3 times. P< 0.05 was deemed to be significant. For all graphs, blue bars represent single knockdowns, purple bars represent double knockdowns, and the orange bar represents triple knockdown.

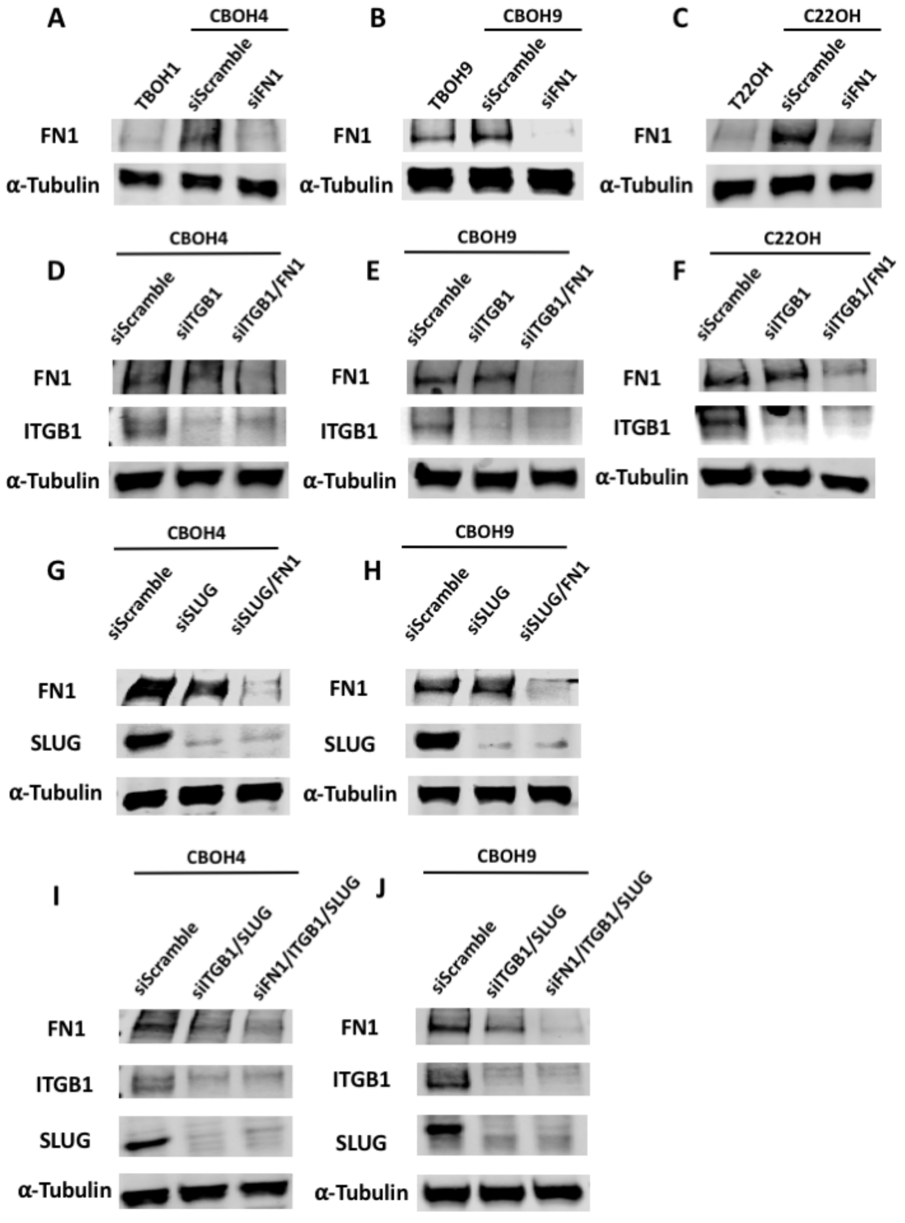

<u>Figure S2:</u> **Specific siRNA-mediated knockdowns of FN1, ITGB1, and SLUG.** Western blots were performed to confirm siRNA transfection success for all appropriate targets- for single, double, and triple knockdowns. **A-C**) Single Knockdown of FN1 in each of our CTC lines. FN1 levels in our primary tumor cell lines are less in comparison to CTCs treated with siScramble. Reduced FN1 expression was observed for siFN1-transfected CTCs. **D-H)** ITGB1, SLUG, and double knockdown siRNA transfection efficiencies were measured in our CTCs. **I-J)** Double and triple knockdown efficiencies for FN1, ITGB1, and SLUG were confirmed in our CTCs.

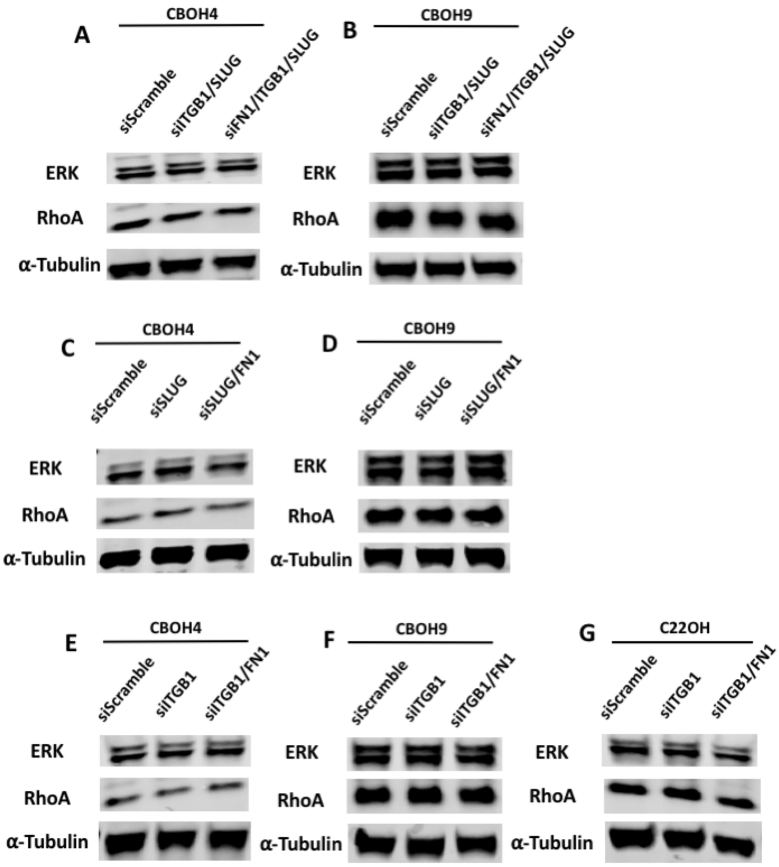

<u>Figure S3</u>: **Confirmation of double and triple knockdown specificity and exclusion of off-target knockdown effects.** Western blots were performed with CTCs subjected to double and triple knockdowns of FN1, ITGB1, and SLUG. ERK and RhoA served as our testable off-targets. **A-B**) CTCs with ITGB1/SLUG double knockdown and FN1/ITGB1/SLUG triple knockdown. **C-D**) CTCs with SLUG/FN1 double knockdown. **E-G**) CTCs with ITGB1/FN1 double knockdown. No expression change is observed for other targets in any double or triple knockdowns, ruling out off-target knockdown effects.

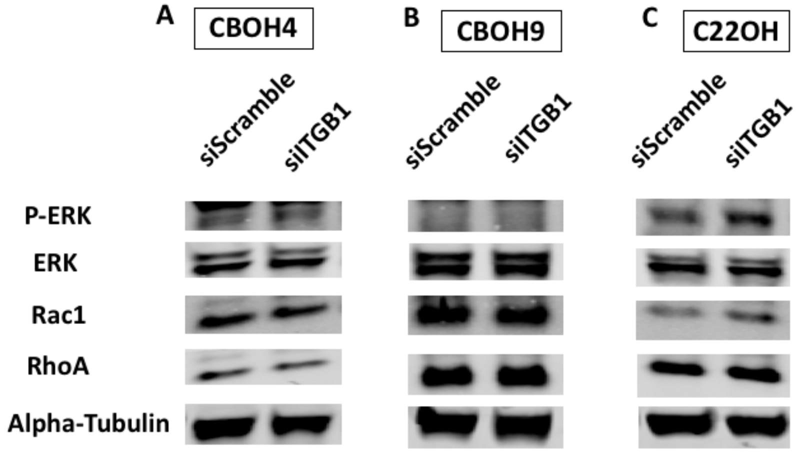

<u>Figure S4</u>: **Knockdown of ITGB1 does not affect P-ERK, ERK, Rac1, or RhoA protein expression.** Western blot analyses of several molecules belonging to common migratory pathways were performed for **A)** CBOH4, **B)** CBOH9, and **C)** C22OH. Expression was normalized against alpha-tubulin. Experiments were carried out at least 3 times.

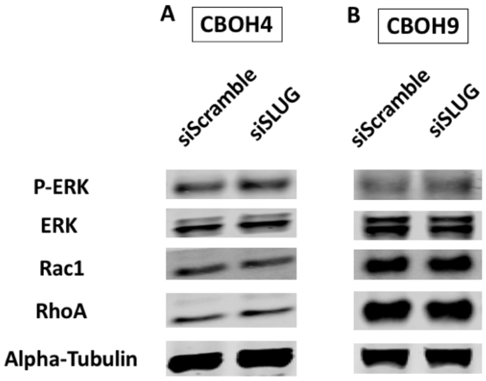

__Figure S5__: **Knockdown of SLUG does not affect P-ERK, ERK, Rac1, or RhoA protein expression.** Western blot analysis of several molecules belonging to different migratory pathways was performed for **A)** CBOH4 and **B)** CBOH9. Expression was normalized against alpha-tubulin. Experiments were carried out 3 times.
